# Measuring Torque and Temperature in a Rotating Shaft Using Commercial SAW Sensors

**DOI:** 10.3390/s17071547

**Published:** 2017-07-02

**Authors:** Diogo Silva, Joana C. Mendes, António B. Pereira, François Gégot, Luís N. Alves

**Affiliations:** 1 Department of Electronics, Telecommunications and Informatics, University of Aveiro, 3810-193 Aveiro, Portugal; diogofsilva@ua.pt (D.S.); nero@av.it.pt (L.N.A.); 2 Instituto de Telecomunicações, Campus Universitário de Santiago, 3810-193 Aveiro, Portugal; 3 Centre for Mechanical Technology and Automation, Department of Mechanical Engineering, University of Aveiro, 3810-193 Aveiro, Portugal; abastos@ua.pt; 4 SENSeOR, 18 rue Alain Savary, 25000 Besançon, France; francois.gegot@senseor.com

**Keywords:** SAW sensor, rotating shaft, torque measurement

## Abstract

Real-time monitoring of torque in a rotating shaft is not easy to implement with technologies such as optic fiber sensors or strain gages. Surface acoustic wave (SAW) sensors are wireless and passive and can be used to monitor strain in moving parts. Commercial solutions (sensors, antennas and interrogation unit) can easily be purchased from some companies; however, they are not customized and may not meet the specificity of the measurements. In order to evaluate the adequacy of commercial off-the-shelf (COTS) solutions, temperature and strain sensors fabricated by SENSeOR (Besançon, France) were mounted on a load cell. The sensors were calibrated using a thermal chamber and a universal testing machine. The load cell was then assembled together with a steel shaft that rotated at different speeds inside an oven. The commercial antennas were replaced with an RF (radio frequency) coupler and the sensors were interrogated with the commercial interrogation unit. The influence of rotation in the accuracy on the measurements, as well as the adequacy of the sensors structure, was evaluated. It can be concluded that SAW sensors can be used to measure temperature or torque in a rotating environment; however, some customization of the components is required in order to overcome the limitations posed by COTS sensing solutions.

## 1. Introduction

Nondestructive testing techniques are widely used for the early detection of potential failure of a material, component or system. These techniques do not alter nor damage the article under inspection and, as such, provide a valuable tool to increase the safety while minimizing the associated costs. However, these inspections are usually prescheduled and do not give information about the real behavior of the components during operation. Real-time structural health monitoring techniques, on the other hand, provide real data obtained during the operation of the system, allowing for the early detection of potential structural failures and the corresponding adoption of mitigating actions before an accident occurs. The predictive maintenance of an industrial system relies, to a large extent, on the possibility of monitoring continuously and in real-time sensors that have been strategically placed in selected components. An example is the measurement of vibrations and forces in machines; in case a pre-established value is reached, the order of repairing is triggered before being reached the state of stop by failure. Furthermore, the new trends and the so-called Industry 4.0 is based on SCADA (supervisory control and data acquisition) systems, which are an integral part of industrial process control and equipment. Sensing elements for parameters such as pressure, flow, temperature or strain, among others, are one of the key elements of such systems.

Strain is usually measured with strain gage transducers. These widespread components are composed of a thin metallic sheet or wire whose electric resistance changes as it is stretched or compressed; this change is then converted into the strain value. However, the use of strain gages to measure strain in a rotating shaft requires the use of slip rings to connect the gages with the required interrogation electronic module; this increases the complexity of the system and may become a noise source, with a negative impact on the accuracy of the required measurements.

Strain can also be measured with fiber-optic sensors using Fiber Bragg gratings. These sensors can be embedded in the structures to be monitored and, when the structure is deformed, the displacement felt by the optic fiber influences the propagation of the light and causes changes in the intensity, phase, polarization or wavelength of the radiation. In the case of Bragg gratings, for instance, the change in the internal reflections at the gratings caused by the fiber displacement can be monitored by light interferometry techniques. These sensors are the preferable solution for remote monitoring or when low electromagnetic interference (EMI) sensitivity is required. However, in the case of monitoring, the deformation of a rotating shaft, the optical signals need to be routed out and optical slip rings are tailored for digital data transfer only, so these sensors are also not suitable for the monitoring of a rotating shaft.

Surface acoustic wave (SAW) technology provides a third technique to monitor the strain that is felt by a mechanical component. An SAW device is composed of a piezoelectric slab with one interdigitated (IDT) contact and some reflectors. When an alternating electric field is applied to the IDT of a SAW resonator, a mechanical deformation of the piezoelectric substrate takes place. If the distance between the IDT fingers is such that the frequency of the electric signal, $f_{c}$, is related with the phase velocity $V_{p}$ and the wavelength of the SAWs λ by $f_{c}=\frac{V_{p}}{\lambda }$, a resonance condition happens and a SAW wave propagates across the surface of the piezoelectric material. SAW waves were described by Lord Rayleigh in the 19th century [1] and are similar to longitudinal seismic waves, in the sense that they only penetrate solids up to a depth of the order of magnitude of the wavelength, they undergo little attenuation as they progress on the surface, and the movement of particles in the solid is elliptical. Propagating velocities in common materials (such as quartz or lithium niobate) are of the order of 10^−5^ of the speed of light. If an external magnitude (such as temperature or deformation) influences the propagation characteristics of the piezoelectric materials, the phase velocity of the SAW waves changes and the frequency $f_{c}$ of the sensor changes accordingly. By probing the resonance frequency of the SAW sensor, the user is able to get an indirect measurement of the parameter under surveillance. The operating frequency of SAW sensors depends on the phase velocity of the acoustic waves and on the dimensions of the IDT fingers, which, in turn, are limited by the photolithographic process. Typical operating frequencies range between 30 MHz and approximately 3 GHz. Commercial devices usually operate in two different bands, 433.92 MHz and 2.45 GHz (ISM bands).

SAW sensors have a few characteristics that make them the ideal choice for measurement of strain on moving parts or in harsh environments. The IDT can be physically connected to an RF antenna and the interrogation of the device can be made remotely, that is, without the need of a wired connection. In addition, the sensors respond with a fraction of the energy sent by the interrogation unit, meaning that they are passive devices that do not require external power or batteries. SAW sensors have other advantages as well. They are low-cost small devices (typically a few square millimeters), exhibit high immunity to EMI and are able to operate under harsh environments. Depending on the operating frequency, the antenna and the RF environment for each particular case, reading distance can be as high as 3 m.

SAW devices have been used in telecommunications for several decades as resonators and filters. More recently they have started to be used as sensors and identification (RFID) tags. Due to their high resilience, SAW sensors have been used to monitor temperature under extreme conditions, such as the inside lining of metallurgical vessels [2], jet engine turbine blades [3] or within the metal-oxide column of surge arresters [4]. Commercial solutions allow real-time and continuous monitoring of temperature in critical electric power transmission and distribution assets [5]. SAW sensors have also been used to measure the cutting forces in computer numerically controlled (CNC) turning [6,7] and strain on rotating shafts [8]. The strain sensitivity of SAW sensors was studied on Chip-level by Hempel and his co-workers [9]. In the automotive industry, these sensors have been used for torque measurement [10,11], in kinetic energy recovery systems [12] and to monitor in real time the tire pressure of mining and commercial [13] and high performance racing vehicles [14]. High acquisition rates of commercial interrogation units enable the use of these components to measure vibration of mechanical structures [15]. Besides sensing capabilities, SAW devices can also behave as RFID tags. Commercial solutions are already available for the railway [16] and metallurgical [17] sectors.

This work intends to evaluate the possibility of using the commercial off-the-shelf (COTS) system fabricated by SENSeOR (Besançon, France) to measure torque and temperature of a rotating shaft. The system is composed of temperature and strain sensors and the corresponding interrogation unit. Since the COTS RF antenna is not appropriate to be used under rotation, a simple RF coupler was designed and fabricated. An aluminum load cell was fabricated and instrumented with the sensors. The temperature sensor was calibrated using a thermal chamber. The strain sensors were calibrated using a mechanical setup designed and fabricated specifically for that purpose. A second mechanical setup was designed to perform the measurements under rotation. The influence of the rotation in the accuracy of the measurements was evaluated at different temperatures and rotation speeds in the absence of external torque. Finally, an external torque was applied to the rotating shaft while the strain sensors were interrogated with the interrogation unit. As a general conclusion, it can be stated that commercial sensors can be used to measure torque and temperature of a rotating shaft. However, the sensors’ response is hampered by some particular characteristics of COTS components that have a negative impact on the accuracy of the complete system. These characteristics have been identified and corrective measurements proposed.

## 2. Materials and Methods

The system that was used in this work is based on a 433 MHz kit fabricated by the French company SENSeOR; the kit includes a temperature sensor, two strain gages and the corresponding interrogation unit.

The sensors were mounted on the surface of a load cell that consisted of a hollow cylinder fabricated in aluminum Al7075-T6. The cell was 90 mm long, inner and outer diameters were 74 and 80 mm, respectively. A flat 24 × 50 mm^2^ surface was machined to allow for the fixation of the sensors. Since the load cell was assembled together with a rotating shaft, the antenna was replaced by a large diameter RF coupler that keeps the sensors in line of sight with the interrogation unit.

### 2.1. SAW Sensors

**Figure 1:**
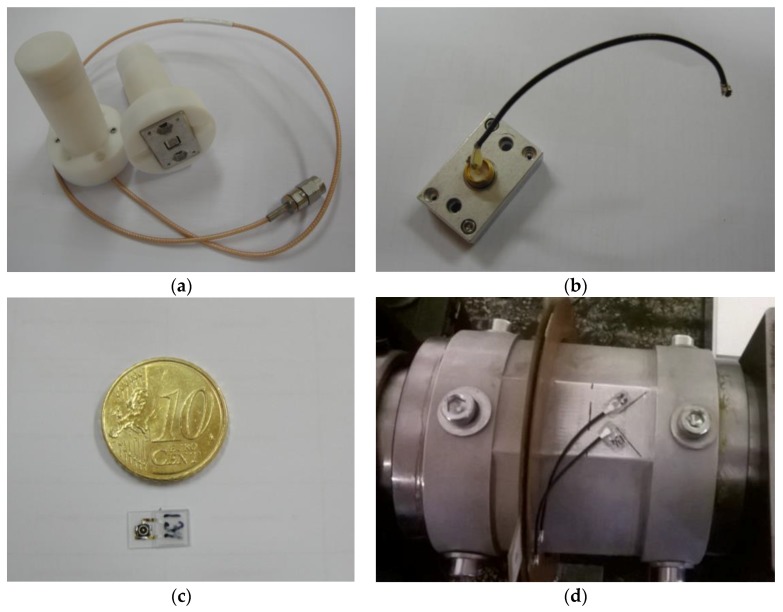
(**a**) COTS commercial off-the-shelf temperature sensor module, composed of sensor chip embedded in an aluminum cage and attached to a radome with the antenna; (**b**) ultra-small surface mount coaxial W.FL cable soldered directly on the SMA (SubMiniature version A) connector connected to the sensor chip embedded in aluminum cage; (**c**) SAW strain gage with W.FL connector clearly visible; (**d**) SAW (surface acoustic wave) strain gages mounted on load cell using half-bridge configuration.

Temperature was measured with a TSE F162 sensor chip (SENSeOR, Besançon, France) mounted in a 5 × 5 mm^2^ SMD (surface-mount device) packaging. This sensor has two resonators with different temperature coefficients. Room temperature (25 °C) resonant frequencies and temperature coefficients for each resonator (taken from the data sheet) can be seen in Table 1. In the COTS solution, the SMD packaging comes embedded in a 26 × 16 × 54.7 mm^3^ aluminium cage that can be glued or screwed directly on the surface of the material to be probed. The weight of the sensor chip and aluminium package is 7 g. The cage is further embedded inside a PTFE (polytetrafluoroethylene) radome 54.8 mm-high with a cylindrical 35.5 mm-diameter base—Figure 1a. The weight of the aluminium cage embedded in the radome is 35 g. Inside the radome, an omnidirectional antenna is connected to the sensor by means of ultra-small surface mount coaxial W.FL connectors (https://www.hirose.com/product/en/products/W.FL/). It is also possible to obtain the sensor module with an additional coaxial cable that can be connected directly to the interrogation unit (Figure 1a). However, this solution is not appropriate for the current application; on one side, the large volume and height of the radome make it inappropriate to be assembled on a rotating shaft; on the other side, the omnidirectional radiation pattern of the antenna does not guarantee a stable communication link between the sensor and the interrogation unit. As such, the radome and antenna were detached from the sensor chip and corresponding aluminium cage and a W.FL cable was soldered to the male 50 Ω SMA connector that is connected to the sensor—Figure 1b. After this step, the sensor (embedded in the aluminium cage) was fixed on the load cell with M-Bond 200 Glue and M-Bond 200 catalyst; thermally conductive paste guaranteed the thermal contact between the sensor chip and the surface of the load cell. The W.FL cable attached to the sensor was then connected (i) directly to the RF coupler or (ii) to the interrogation unit or to a dipole antenna (for calibration purposes) with an SMA (SubMiniature version A) coaxial cable.

After being mounted on the load cell surface, the temperature sensors were calibrated using a FITOTERM 22E thermal chamber (Aralab, Lisbon, Portugal) with the sensors and interrogation unit connected directly to dipole antennas. The load cell was exposed to temperature sweeps, with 5 °C resolution, from 20 °C to 80 °C. Between each temperature step, there was a time interval of 45 min; a J-type thermocouple digital thermometer was used to measure the temperature directly on the load cell surface. At each step, the response of the sensors was recorded using the interrogation unit.

Torque was measured with SENSeOR SSE E015 and SSE E017 strain gages. The thickness of each gage is 350 µm, while the length and width are 9.0 and 5.5 mm, respectively, and the weight is ≈0.04 g. Figure 1c shows the detail of one of the gages with a clear view of the W.FL connector. Each strain gage consists of one resonator with a different resonant frequency—Table 1. The gages were fixed on the load cell surface using M-Bond 200 Glue and M-Bond 200 catalyst. In order to measure applied torque, a half-bridge configuration was used with the strain gages placed at a 45° angle with the load cell neutral axis—Figure 1d. The W.FL cables connected to each of the gages were connected (i) directly to the RF coupler or (ii) to the interrogation unit with an SMA coaxial cable.

As can be seen from Table 1, the resonant frequencies do not overlap and simultaneous interrogation can be performed without the risk of spectral overlap.

### 2.2. Interrogation Unit

The sensors were interrogated by the SENSeOR wideband radio-frequency transceiver WR D005. This unit operates in the [430.5, 449.5] MHz frequency band. It provides digital RS232/USB and analogue outputs. The maximum sampling rate/sensor is, according to the data sheet, 150 Hz (depending on the application). The dimensions of the interrogation unit are 18.4 × 10.9 × 3.0 cm^3^.

Once the interrogation unit is turned on, the user is prompted to choose a configuration. Different types of measurements are possible: quarter-bridge, half-bridge, half-bridge with temperature compensation or temperature alone. After the configuration has been chosen, the user may configure the filtering and recording parameters and correct the frequency offset.

**Figure 2:**
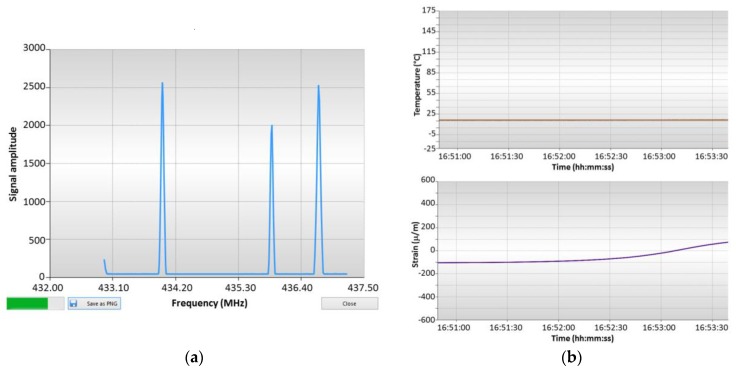
(**a**) “Oscilloscope” mode of interrogation unit, showing the response of sensor SSE E017 and the two resonance peaks of sensor TSE F162; (**b**) typical temperature and strain output windows.

After this configuration, the user can start acquiring data. The “Oscilloscope” mode is similar to the display of a spectrum analyzer, where the frequency response of the sensors is shown. As an example, Figure 2a shows the response of sensor SSE E015 (peak centered at 433.95 MHz) and the two resonance peaks of sensor TSE F162 (435.88 and 436.58 MHz). The left most resonance is related with the response of a 3rd sensor and falls outside the visible window. Typical temperature and strain output windows are depicted in Figure 2b.

### 2.3. RF Couplers

A dipole antenna is the simplest solution for establishing communication at 434 MHz band. However, in the present case, this solution would compromise the line of sight, so a large diameter microstrip coupler [18] was implemented.

**Figure 3:**
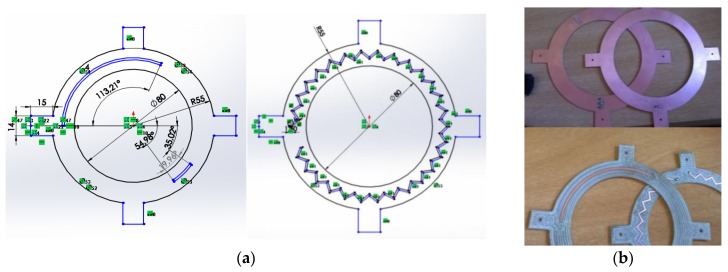
(**a**) Diagrams of rotor coupler line (longer line) and stator zigzag-shaped coupler line; (**b**) fabricated couplers.

The stator and rotor couplers were fabricated using FR-4 epoxy laminate. This material has a copper conductive layer and a dielectric constant of 4.5 and is available in plates of different thickness. On the top layer, micro strip transmission lines were patterned by selectively removing the copper layer, while the back surface remained covered with a copper layer, behaving as a ground plane. The length of the stator line was chosen to meet one wavelength $L_{S}=\lambda$—under these conditions, the travelling wave regime minimizes the amplitude variation of the signal along the stator circumference. The length of the rotor line was LR=λ4. The possible combinations for the length *L* and width *W* of the stator and rotor lines were calculated as a function of the thickness *W* of commercially available FR-4 substrates using TX-Line by AWR transmission line design tool—Table 2. *L* remains almost unchanged while *W* changes significantly with the thickness of the FR-4 substrate. To keep the largest *L/W* ratio, FR-4 substrate with a thickness of 0.8 mm was chosen. The inner diameter of the couplers was ≈80 mm (to fit the external diameter of the load cell), which corresponds to a minimum perimeter of ≈250 mm, and the outer diameter was 110 mm. The λ4 rotor line was obtained with a curved line while the stator line had a zigzag shape (Figure 3a). The fabricated couplers can be seen in Figure 3b.

The stator coupler was fixed with screws to an L-shape metallic piece mounted directly on the table; the transmission line was terminated with 50 Ω and was connected to the interrogation unit with a coaxial cable. The rotor coupler was mounted directly on the shaft. The rotor was designed in a way that its inner diameter meets the external diameter of the load cell. After inserting the shaft through the coupler’s hole, it was further fixed to the load cell with epoxy. This guarantees that shaft and rotor coupler behave as one single body once the system is rotating. The transmission line was short-circuited and was connected to the SAW sensors with W.FL cables. The scattering parameters of the implemented couplers (Figure 3b) were measured with a PNA E8361C network analyzer (Santa Rosa, CA, USA).

### 2.4. Calibration Setup

**Figure 4:**
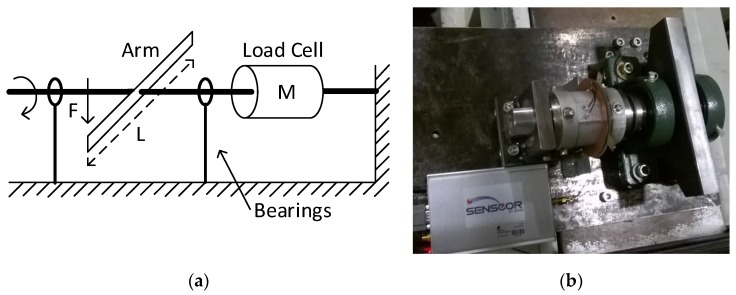
(**a**) Simplified schematic representation of the calibration setup; (**b**) detail of the load cell, strain sensors and moveable arm.

To determine the gage factor $S_{G}$ of the SAW strain gages, a static calibration setup was previously designed and implemented. The operation principle is represented in Figure 4a. A force $\vec{F}$ is applied perpendicularly to a metallic arm that is connected to a steel shaft. The load cell is attached to this shaft, which, in turn, stands on two bearings. Under these conditions, the torque *M* applied to the load cell is given by the following equation:

(1) $$M=F\cdot \frac{L}{2}\cdot \sin\theta ,$$

where *F* is the applied force, *L* is the length of the moveable arm and *θ* is the angle formed between the applied force vector and the arm. Since the force is applied perpendicularly to the moveable arm and the maximum vertical displacement of the arm is negligible when compared to its length, for practical considerations sin(θ)≈1 and this term can be neglected.

The instrumented load cell was assembled in the calibration setup and the RF couplers were positioned with a relative orientation of 0°. A Shimadzu Autograph AGS-10kN Universal Testing Machine (UTM) (Kyoto, Japan) applied strain in the downward direction in the moveable arm; measurements were performed at room temperature. The values of applied strain varied between 0 and 47 µm/m with a step of 4.7 µm/m. The procedure was repeated afterwards but with strain applied in the upward direction using a steel cable connected to the arm. For each applied strain, the response of the gages was measured with the interrogation unit. The detail of the load cell, the strain sensors and the moveable arm can be seen in Figure 4b.

### 2.5. Setup for Dynamic Measurements

**Figure 5:**
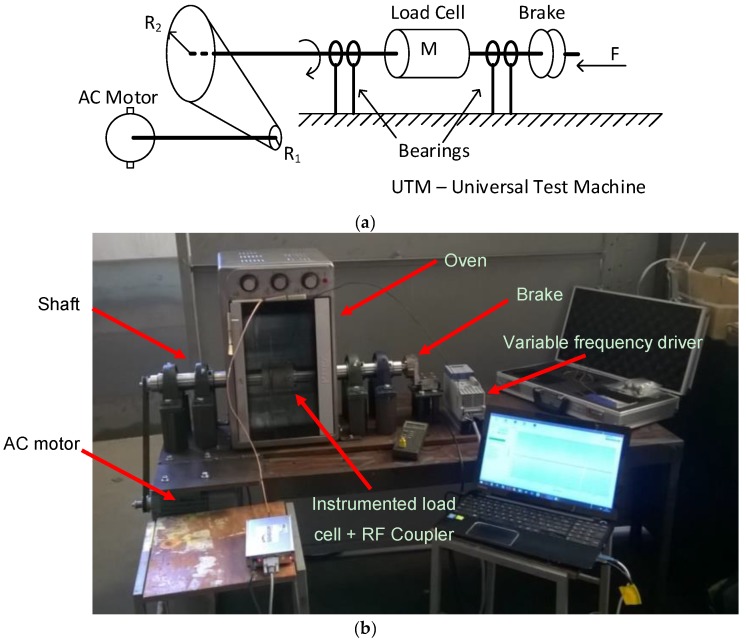
(**a**) Simplified schematic representation of the setup used for measurements under rotation; (**b**) setup assembled for tests under rotation.

Figure 5a represents the schematic diagram of the setup for making the measurements under rotation. An alternating current (AC) motor is connected to a steel shaft through a couple of pulleys and a toothed belt. The load cell is attached to this shaft, which, in turn, stands on two bearings. Finally, a brake is connected to the free side of the shaft.

Considering that the loading of the AC motor by the shaft is negligible, the torque felt by the load cell is equal to the torque produced by the brake. When the brake is pressed by a force $\vec{F}$ parallel to the shaft axis, the generated torque *M* is equal to:

(2) $$M=\frac{2}{3}\cdot \mu \cdot \left|\vec{F}\right|\cdot R,$$

where μ is the friction coefficient between the brake disk and the shaft, $\left|\vec{F}\right|$ is the absolute value of the applied force and R is the radius of the shaft. For the present case, the friction coefficient was μ=0.3.

Figure 5b shows the assembled setup. To control the ambient temperature, the shaft with the attached load cell penetrated a commercial oven perforated for this effect. The shaft was mounted on two mechanical bearings and the stator antenna was fixed to the oven by a metallic L-shaped piece. A motor NORD SK80-LH/2 (Oliveira do Bairro, Portugal), with a nominal rotating speed of 2825 rpm, was connected to the shaft with a toothed belt. The speed of the motor was controlled with a frequency inverter NORDAC AC Vector Drive (Oliveira do Bairro, Portugal) of the SK500E family, with 200–240 V 3-phased power supply. The rotation speed of the shaft was measured with a digital tachometer HS2234. The braking system consisted of a metallic brake pad that was pressed perpendicularly against the shaft to reduce the rotation velocity. A load cell was used to measure the force impressed on the braking system. Whenever necessary, the temperature in the air surrounding the load cell was measured with a J-type thermocouple digital thermometer.

This setup was used for three purposes: (i) to measure the temperature response of the strain gages in the absence of applied torque; (ii) to record the response of the temperature sensor at different rotation speeds and (iii) to measure the applied torque under rotation. For task (i), the RF coupler was positioned with a relative orientation of 0°. The resonant frequencies of each gage were measured under static conditions, without any applied external torque, between 0 and 80 °C with a 10 °C interval. The resonant frequencies were also measured at the reference temperature 25 °C. To guarantee the uniform heating of the cell, each heating cycle was followed by a 1 h waiting period. For task (ii), the measurements were performed at three different temperatures (25, 47.5 and 60 °C), with rotation speeds between 0 and 2300 rpm with increments of ≈300 rpm in both rotation directions and without applied torque. For each experimental condition, 1000 readings of the resonant frequencies of the temperature and strain sensors were recorded. The average resonating frequencies and corresponding standard deviations were then computed from the experimental data. In order to improve the accuracy of the measurements, the maximum standard deviation allowed to accept an interrogation cycle data frame was 3 kHz and 22 frequency readings were averaged to extract a data point. For task (iii), the tests were performed at room temperature and at a rotation speed of ≈150 rpm. The force applied to the brake varied between 0 and 2000 N, with steps of 250 N. The data were recorded during ≈10 s, to avoid the heating of the braking pad.

## 3. Results

### 3.1. RF Couplers

**Figure 6:**
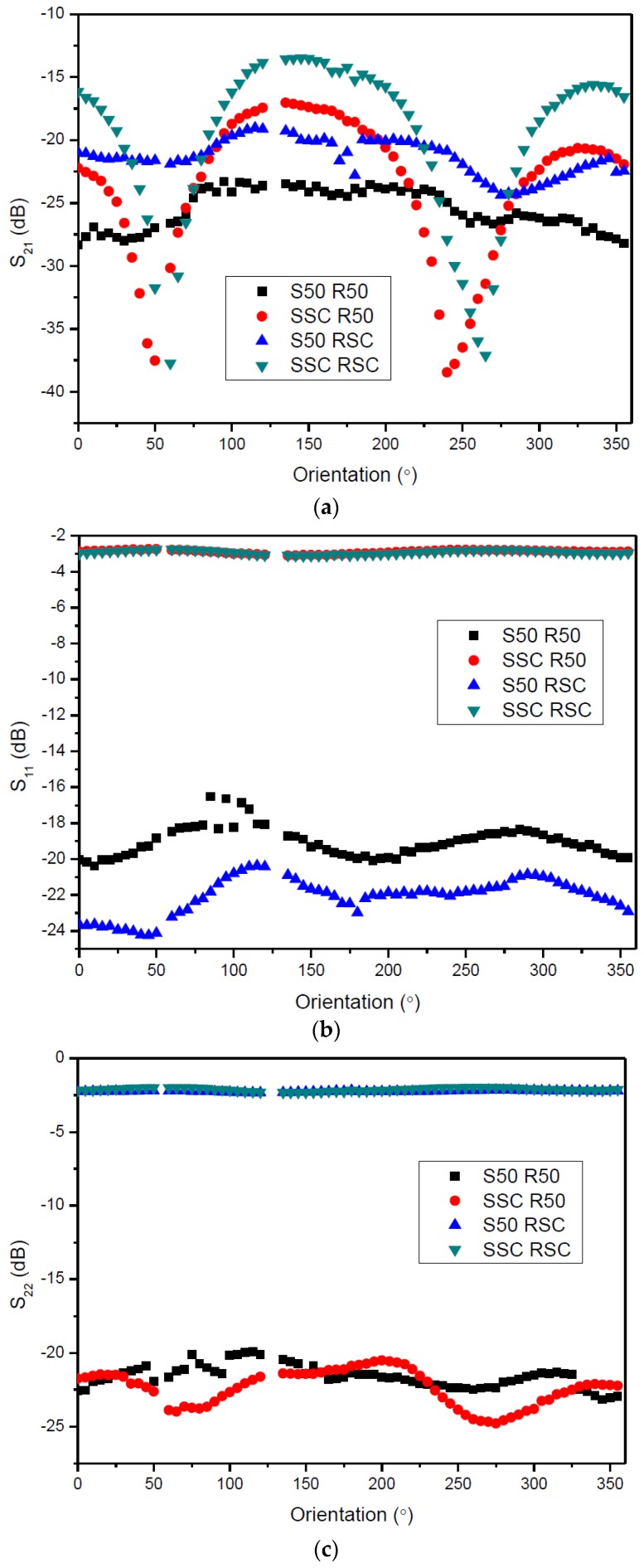
(**a**) S_21_, (**b**) S_11_ and (**c**) S_22_ measured for different stator/rotor terminations and coupler orientations.

The S parameters (S-parameters are used for the characterization of multi-port networks in the frequency domain. S-parameters are a measure of the reflected and transmitted energy arriving at each port of the network. In this sense, S-parameters encompass reflection coefficients as measure of each port impedance and transmission as a measure of the coupling between ports. Interested readers are referred to [19]) of the RF couplers at 433 MHz were measured with different terminations (50 Ω and short-circuit) at different couplers relative orientation—Figure 6. The introduction of a 50 Ω resistor in the second port of the stator coupler flattens the S_21_ for all orientation angles (Figure 6a). On the other hand, short-circuit termination creates periodic negative peaks in the transmission response that repeat every L=λ4 (with *L* the length of the stator micro strip line). The influence of the termination of the rotor coupler is not so critical and best results are obtained with the rotor in short-circuit. The termination with a 50 Ω load deteriorates S_21_ in around 5 dB in comparison with short-circuit termination; however, it does not compromise the flatness of the response for all coupler orientations. Figure 6b shows S_11_ for different terminations and orientation angles. When the stator is terminated with a short-circuit, the reflection at the stator is maximum, which means that the energy transferred to the rotor is minimum. On the other hand, termination of the stator with 50 Ω load decreases the reflection and maximizes the energy transferred to the rotor. Again, best results were obtained with the rotor in short-circuit. Figure 6c shows the S_22_ for different terminations and orientation angles. The minimization of the reflected energy at the rotor side happens when the rotor is terminated with a 50 Ω load. The termination of the stator has little influence in the S_22_.

Following the results obtained with this characterization, the stator was terminated with a 50 Ω load and the rotor was kept in short-circuit. This minimizes the reflections at the stator port and guarantees the maximum flatness of the transmission with the rotation angle, which, in turn, reduces the parasitic frequency shift due to the angular variations of the coupling.

A similar coupler was implemented by Boccard and his co-workers [20]; however, they used a coplanar strip line coupler, the coupling elements were de-tuned and resonated at a higher frequency than the SAW device and the design approach aimed at minimizing the frequency pulling and providing 360° angular coverage of the sensor visibility by the reader. This strategy showed better stability of the transmission loss for different angular positions. In our case, both stator and rotor lines were matched to the SAW devices and the transmission loss obtained with the stator matched to 50 Ω and the rotor in short circuit (Figure 6a) is similar to the transmission loss Boccard et al. obtained (Figure 10, [20]).

### 3.2. Calibration of Temperature Sensor

The dependence of the resonant frequency $f_{R_{i}}$ of each resonator with respect to temperature is given by the following expression:

(3) $$f_{R_{i}}\left(T\right)=f_{R_{i}}\left(T_{0}\right)\cdot \left[1+\;C_{1i}\cdot \left(T-T_{0}\right)+C_{2i}\cdot {\left(T-T_{0}\right)}^{2}\right],$$

where $f_{R_{i}}\left(T_{0}\right)$ is the resonant frequency at 25 °C and $C_{1i}$ and $C_{2i}$ are the first and second order temperature coefficients, respectively, of each individual resonator (i=1,2). The temperature *T* is related with the difference between the two relative resonance frequencies $∆f_{r}$ by:

(4) $$∆f_{r}=\frac{f_{R_{1}}\left(T\right)}{f_{R_{1}}\left(T_{0}\right)}-\frac{f_{R_{2}}\left(T\right)}{f_{R_{2}}\left(T_{0}\right)}=\left(C_{11}-C_{12}\right)\cdot ∆T+\left(C_{21}-C_{22}\right)\cdot ∆T^{2},$$

where $∆T=T-T_{0}$ and $T_{0}=25\;{}^{\circ}C$. Since $C_{11}$ and $C_{12}$ have opposite signs and $C_{21}$ and $C_{22}$ have the same sign, the differential measurement enhances the whole sensor sensibility to temperature and the output linearity significantly while the quadratic dependence is decreased.

**Figure 7:**
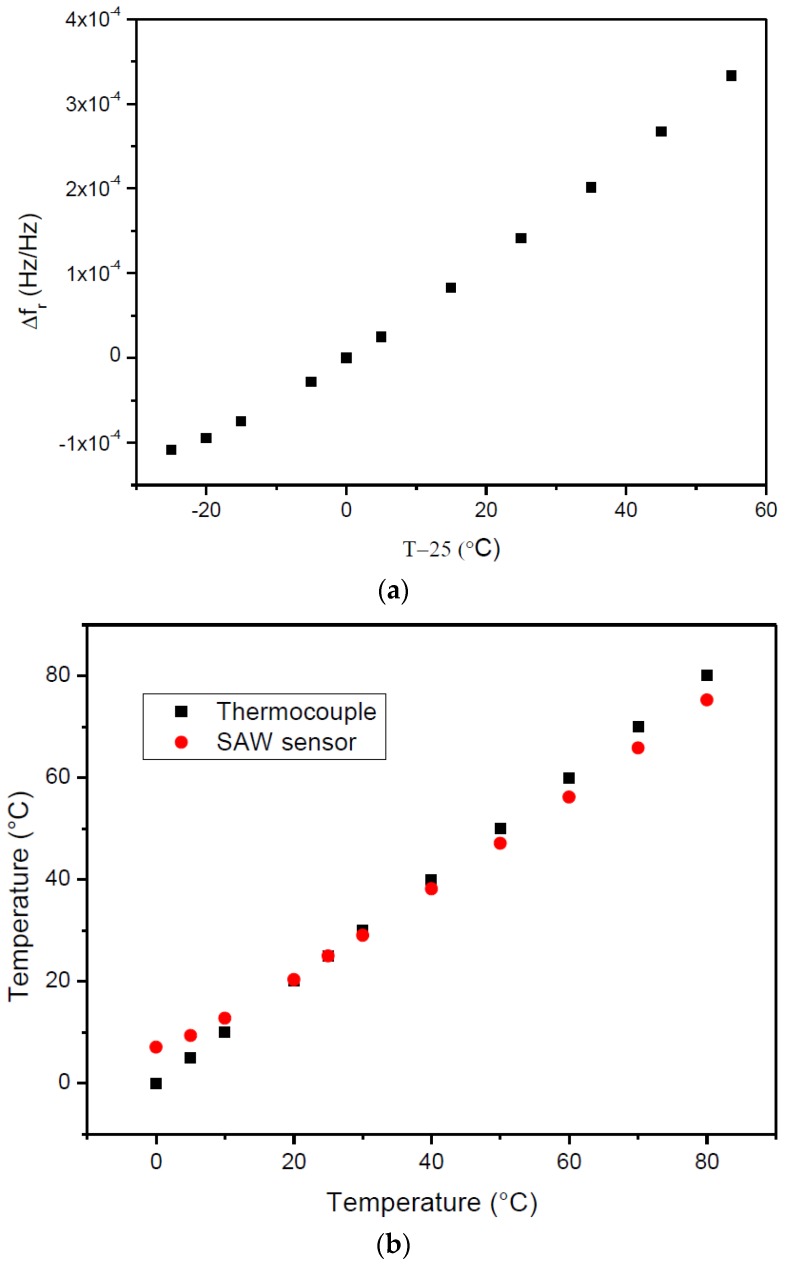
(**a**) $∆f_{r}$ obtained for different ∆T. Two dipole antennas were connected directly to the sensor and interrogation unit, respectively. (**b**) Temperature obtained by the thermocouple (black squares) and SAW sensor (red circles).

Once $∆f_{r}$ for a given temperature is known, the calculation of T with Equation (4) is straightforward. However, the behavior of the temperature sensor mounted in the load cell may not be accurately described using the temperature coefficients taken from the sensor’s datasheet and, as such, the calibration of the temperature sensor was required. The instrumented load cell was placed inside the thermal chamber (as described in Section 2.1), the sensors and the interrogation unit were connected to dipole antennas and the response of the temperature sensor was recorded at different temperatures. The difference between the relative resonant frequencies with temperature is plotted in Figure 7a. The data were fit to a second order polynomial, allowing for the determination of the first and second order temperature coefficients $C_{1}=5.06\times 10^{-6}/℃$ and $C_{2}=18.9\times 10^{-9}/{℃}^{2}$ (Equation (5))—Table 3 (the values obtained with Equation (4) and the theoretical parameters taken from the manufacturer datasheet are included for comparison purposes). Once the coefficients $C_{1}$ and $C_{2}$ are determined, the temperature can be directly calculated applying the quadratic formula to solve Equation (5):

(5) $$∆f_{r}=C_{1}\cdot ∆T+C_{2}\cdot ∆T^{2}.$$

The temperature readings obtained by the thermocouple and the SAW sensor are plotted as a function of the temperature read by the thermocouple in Figure 7b. As temperature decreases or increases below or above ambient temperature, respectively, there in an increasing error between the values measured by the SAW sensor and the thermocouple. This effect is related with the difficulty in obtaining the accurate value of the temperature at the surface of the cell with the thermocouple.

Following this calibration, the sensors and the interrogation unit were connected to the couplers and the calibration procedure was repeated. The extracted coefficients can be seen in Table 3. The theoretical coefficients, obtained by replacing the theoretical coefficients given in the sensor datasheet (Table 1) in Equation (4), are included for comparison.

### 3.3. Calibration of SAW Strain Gages

The gages were fixed on the load cell surface using half-bridge configuration with the gages placed at a 45° angle with the load cell neutral axis in accordance to what is specified in the manual by SENSeOR. With this configuration, the strain felt by each gage $\varepsilon_{1}=-\varepsilon_{2}$ is given by:

(6) $$\varepsilon_{1}=-\varepsilon_{2}=\frac{M\cdot R}{2\cdot J\cdot G},$$

where *M* is the torque, *R* is the radius of the cylinder, *J* is the quadratic moment and *G* is the shear modulus.

Similarly to what happens with temperature sensors, the resonant frequency of the SAW gages is given by:

(7) $$f_{R_{i}}\left(T,\varepsilon \right)=f_{R_{i}}\left(T_{0}\right)\cdot \left[1+\;C_{1i}\cdot \left(T-T_{0}\right)+C_{2i}\cdot {\left(T-T_{0}\right)}^{2}+S_{G}\cdot \varepsilon \right],$$

where $f_{R_{i}}\left(T_{0}\right)$ is the resonant frequency at 25 °C, $C_{1i}$ and $C_{2i}$ are the first and second order temperature coefficients, respectively, of each individual resonator, and $S_{G}$ is the gage factor (i=1,2) and *ε* is the strain felt by each strain gage. The difference between the relative resonating frequencies of each sensor $∆f_{r}$ is given by:

(8) $$∆f_{r}=\frac{f_{R1}\left(T\right)}{f_{R1}\left(T_{0}\right)}-\frac{f_{R2}\left(T\right)}{f_{R2}\left(T_{0}\right)}=\left(C_{11}-C_{12}\right)\cdot ∆T+\left(C_{21}-C_{22}\right)\cdot ∆T^{2}+2\cdot S_{G}\cdot \varepsilon .$$

If the values of the temperature coefficients are the same for both gages, the differential output removes the dependence of the temperature:

(9) $$∆f_{r}=\frac{f_{R1}\left(T\right)}{f_{R1}\left(T_{0}\right)}-\frac{f_{R2}\left(T\right)}{f_{R2}\left(T_{0}\right)}=2\cdot S_{G}\cdot \varepsilon .$$

By substituting Equation (6) into Equation (9) and considering that the quadratic moment of the load cell is given by $J=\frac{\pi \cdot R^{4}}{2}$ (with R the cell radius), torque at a given temperature T can be calculated from the difference of the relative resonating frequencies using the following expression:

(10) $$M=\frac{\pi \cdot R^{3}\cdot G}{2\cdot S_{G}}\cdot ∆f_{r}.$$

The calculation of the torque is thus straightforward once the sensors response and gage factor are known. In practice, however, the measurements are not totally independent of temperature. Differences in the process of gluing the sensors on the load cell surface may introduce asymmetries in the coefficients and, as a result, the resonant frequencies depend on temperature:

(11) $$∆f_{r}=C_{2}\cdot ∆T^{2}+C_{1}\cdot ∆T+2\cdot S_{G}\cdot \varepsilon ,$$

and Equation (10) becomes:

(12) $$M=\frac{\pi \cdot R^{3}\cdot G}{2\cdot S_{G}}\cdot \left(∆f_{r}-C_{2}\cdot ∆T^{2}-C_{1}\cdot ∆T\right).$$

As a consequence, the accurate calculation of the torque applied to the cylindrical load cell also requires the knowledge of the coefficients $C_{1}$ and $C_{2}$. Therefore, before the strain gage was determined, the response of the gages was measured as a function of the cell temperature.

#### 3.3.1. Calibration with Temperature

**Figure 8:**
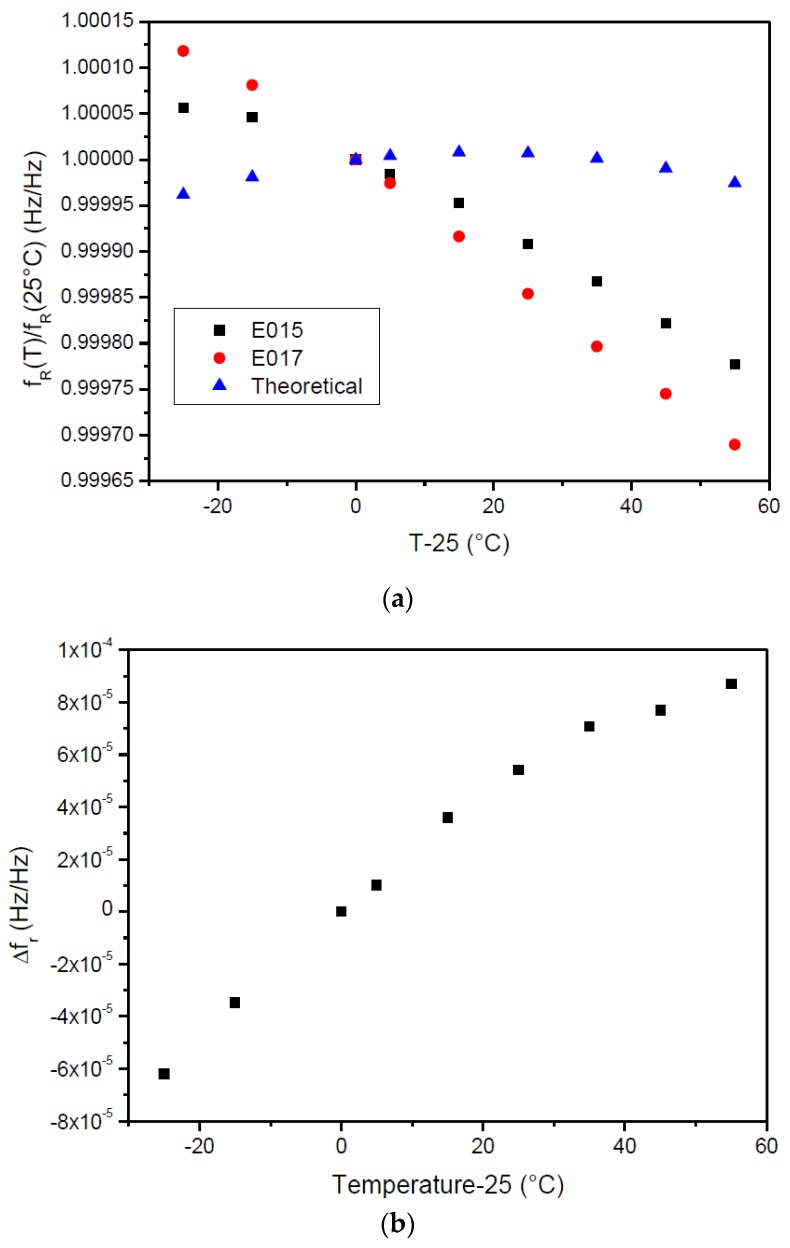
(**a**) Relative resonant frequency of gages SSE15 and SSE17 for different temperature values ∆T; (**b**) $∆f_{r}$ obtained for different temperature values ∆T.

The relative resonant frequencies of SSE E015 and SSE E017 gages measured at different temperatures and in the absence of applied torque are plotted in Figure 8a. The theoretical resonant frequencies are included for comparison (blue triangles). The difference between theoretical and experimental data is quite clear. The difference between the relative resonant frequencies of each gage with temperature is plotted in Figure 8b. Following the same procedure that was used for the calibration of the temperature sensor, the data was fit to a second order polynomial, allowing for the determination of coefficients $C_{1}=2.31\times 10^{-6}$/°C and $C_{2}=-1.38\times 10^{-8}$/°C^2^ (Equation (11)). These coefficients will be used for the calculation of the torque (Equation (12)).

#### 3.3.2. Determination of Gage Factor

**Figure 9:**
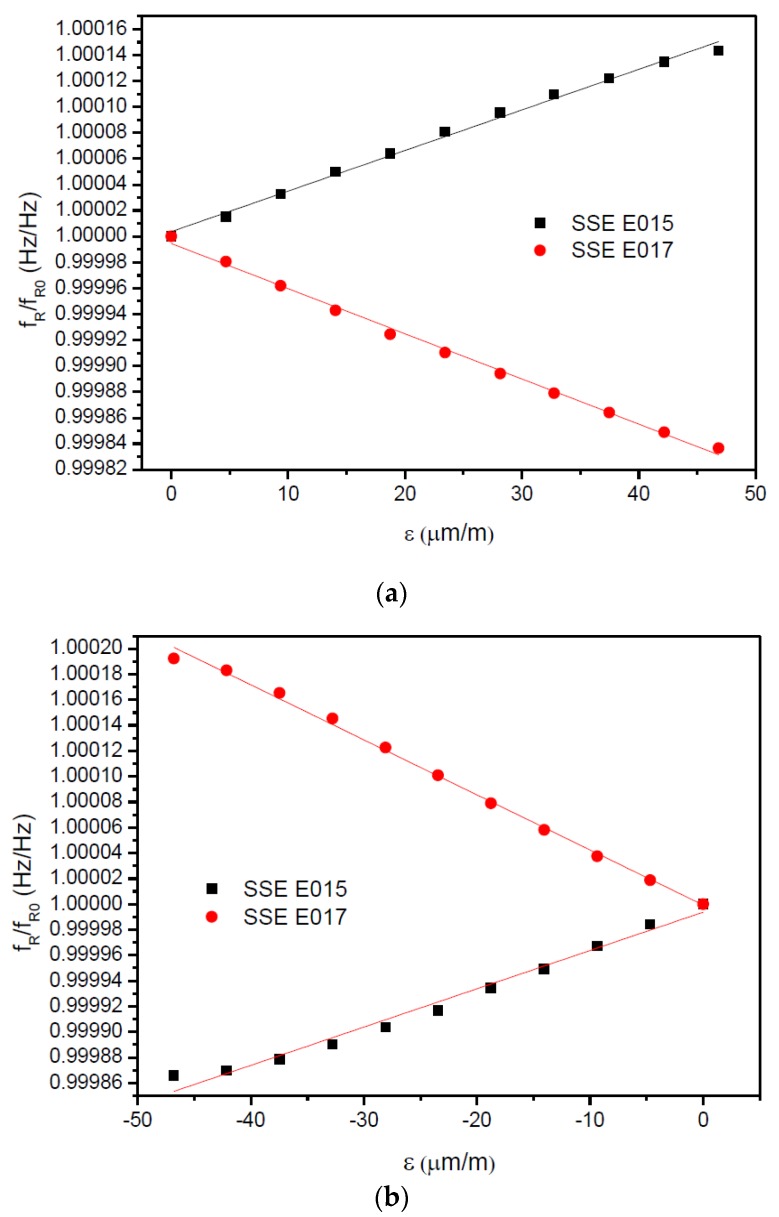
Relative resonance frequency of gages SSE E015 and SSE E017 for different values of strain applied in (**a**) downward direction and (**b**) upward direction. Values were measured at 25 °C.

Figure 9a,b show the individual relative resonant frequencies of both gages against the applied strain in the downward and upward directions, respectively, measured at 25 °C. The gage factors determined with Equation (7) of the individual gages can be seen in Table 4.

**Figure 10:**
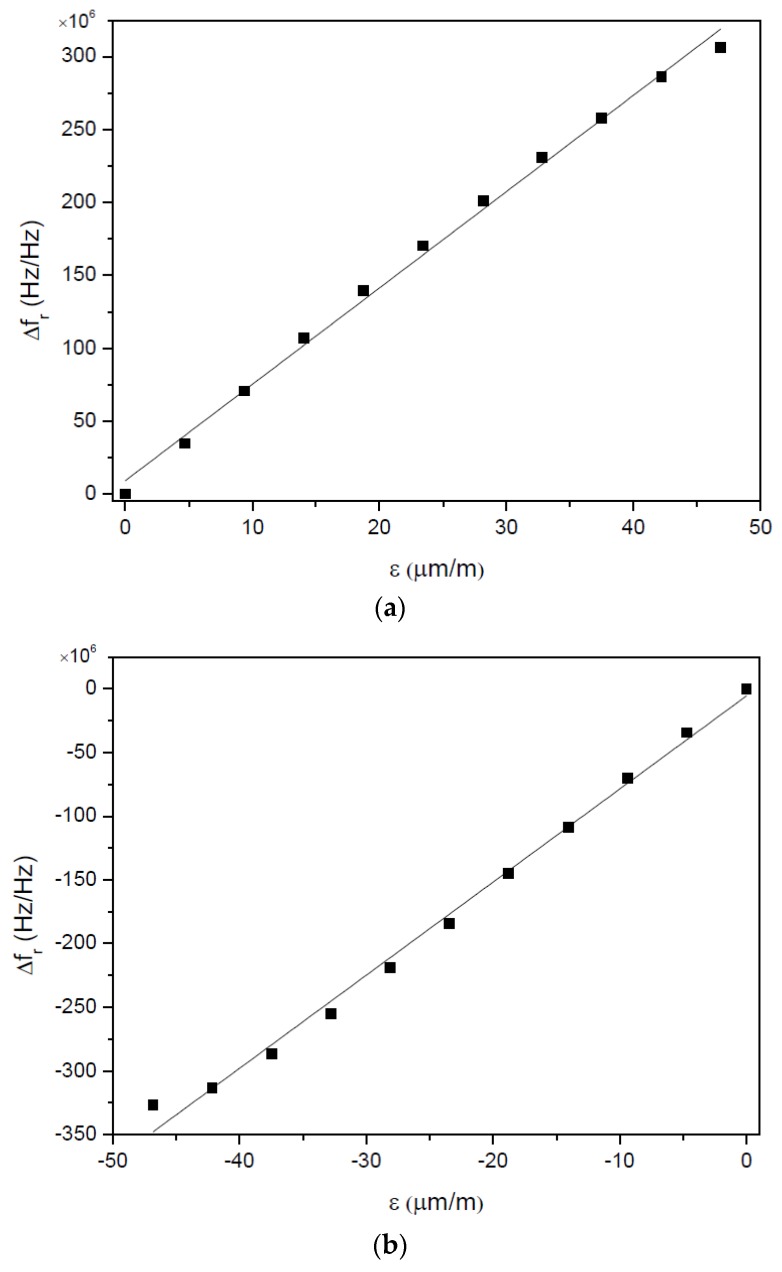
Difference between relative resonant frequencies of gages SSE E015 and SSE E016 for different values of strain applied in (**a**) downward direction and (**b**) upward direction. Values were measured at 25 °C.

Figure 10a,b show the difference between the relative resonant frequencies of both gages against the applied strain in the downward and upward directions, respectively, measured at 25 °C. The gage factor of the load cell (determined with Equation (11)) is $S_{G}=3.30$ for strain applied in the downward direction and $S_{G}=3.66$ with strain applied in the opposite direction.

The gage factors of both sensors are larger than 3. Since the gage factor of a metallic extensometer is typically 2, systems that use SAW sensors may have enhanced accuracy and precision. However, the gage factor obtained with the different sensors and when the force is applied in opposite directions varies by a factor that can be as high as 1.4. Several factors may originate this effect. On one side, the existence of hysteresis associated with mechanical assembly at the UTM may contribute partially to this difference. On the other side, the asymmetries of the manual gluing process may also explain the slight variation of individual gage factors (for instance when the force is applied in the downward direction, 3.13 against 3.48). However, when the force is applied in the upward direction, the individual gage factors are considerably different (3.00 and 4.31). Even if the eventual existence of hysteresis related with the mechanical system may influence the determination of the gage factor, this large difference can only be explained if the process of gluing the sensors influences their response. While this effect may also take place when strain gages are used, it becomes more problematic with SAW strain gages. An extensometer is composed by a metal wire or film attached to a low Young’s modulus carrier film like polyimide or kapton. The Young’s modulus of the carrier film and the glue used to mount the sensors are similar and any strain or deformation in the aluminum is easily transferred to the gage and the asymmetry in the gluing of the gages has no visible effects. On the other hand, SAW strain gages are fabricated in quartz, which has a Young’s modulus similar to that of aluminum (76 GPa for YX and ST cuts [21] against 71.7 GPa for Al7075-T6 aluminum alloy) and, as a consequence, the strain transfer from the aluminum load cell to the piezoelectric material depends on the viscoelastic properties of the adhesive that was used to glue the sensors [22]. Since the gluing of the sensors on the surface of the load cell was made manually, the asymmetries that naturally arise from this process may impact the overall strain transfer and correspondingly the gage factor of the sensors. However, more work is required in order to evaluate the real impact of the adhesive used, as well as to determine if the difference between the gage factors obtained in different conditions can be attributed to the combined effect of the mechanical hysteresis of the system together with the influence of the adhesive in the measurements.

### 3.4. Measurements under Rotation

When the antennas are replaced by the RF couplers, a parasitic passive component (either inductor or capacitor) is introduced in the link budget. When the measurements are made under static conditions, the effect of this parasitic component is not noticed; however, if the measurements are made under rotation, the influence of this component in the link budget may cause introduce some frequency pulling. This effect cannot be accurately accounted for because it would require the knowledge of the couplers coupling parameters at the exact moment the interrogation unit is acquiring the data. Thus, in order to evaluate the impact of the rotation on the accuracy of the measurements, the statistical procedure described in Section 2.5 was applied.

#### 3.4.1. Influence of the Rotation in the Temperature Measurements

**Figure 11:**
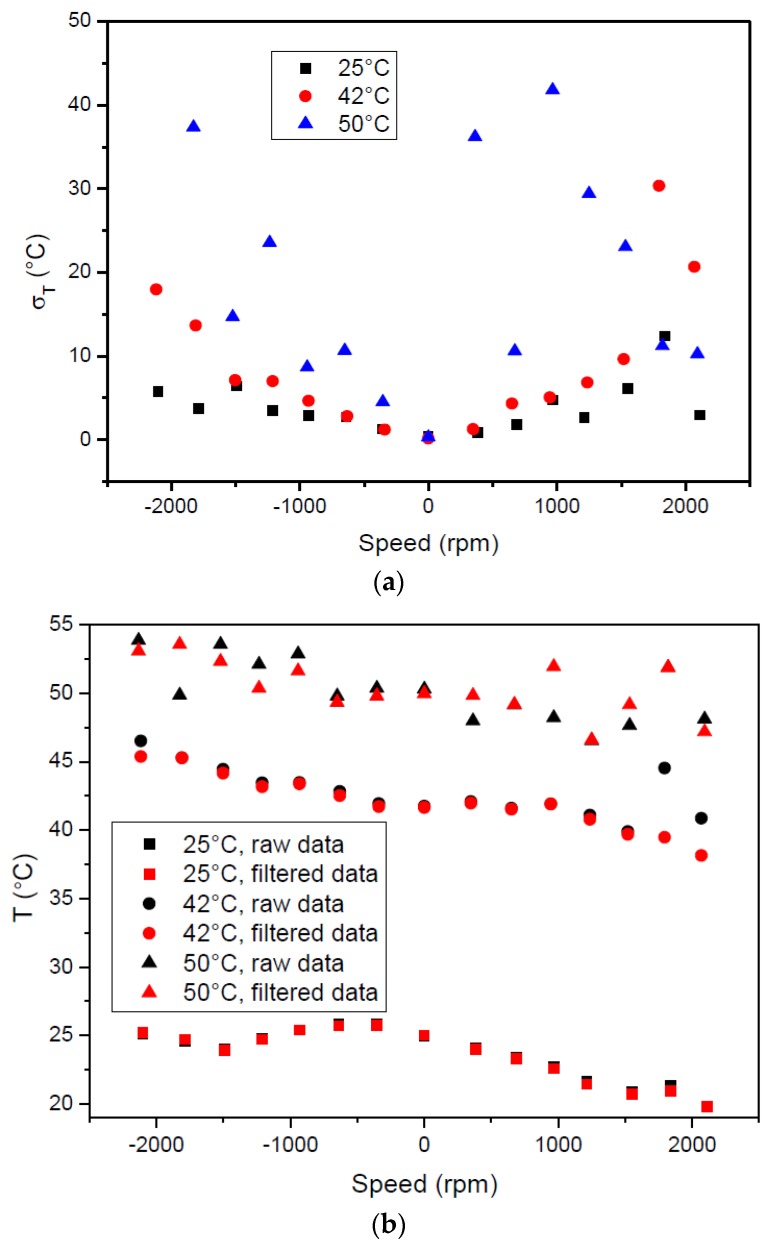
(**a**) Standard deviation and (**b**) temperature measured at different rotation speeds. Black data points: values calculated with all frequency readings. Red data points: values calculated with frequency readings that fall within the standard deviation.

Like what was described in Section 2.5, for each rotation velocity and temperature condition, 1000 frequency readings were recorded and the corresponding temperature was determined using Equation (5) and the coefficients determined during the calibration of the sensor. The thermocouple measured the temperature of the air surrounding the load cell in the three tests performed at 25 (ambient temperature), 47.5 and 60 °C; the temperature measured by the temperature sensor was 25, 42 and 50 °C, respectively. This large error is related to the difference in the temperature of the air surrounding the load cell and the cell itself. The standard deviation of the arrays of temperature readings is shown in Figure 11a. The deviation shows a clear tendency to increase with rotation velocity at 25 and 42 °C, reaching a maximum value of ≈12.4 and 30.4 °C, respectively. This effect is related with the lack of mechanical stability of the implemented stator coupler. Once the shaft rotates, the coupler rotates at the same speed; however, FR4 is not a rigid material and, as such, it also vibrates in a direction paralel to the shaft axis. In addition, the centrifugal force felt by the W.FL coaxial cable is also transmitted to the rotor coupler, which compromises the parallelism of the couplers ever further. Once this happens, the distance between the stator and rotor couplers changes and so does the electromagnetic coupling. For measurements at ≈1800 rpm clockwise, the deviation shows a sudden increase, more evident at 42 °C—this may be due to a mechanical resonance condition that further compromises the electromagnetic coupling. For measurements at 50 °C, however, the deviation shows an erratic behavior, reaching the maximum value of ≈41.8 °C. It should be mentioned that the operating temperature range of the epoxy that was used to glue the temperature sensor decreases from 95 °C in the short term to 65 °C in long-term operation. At 42 °C, the behavior of the epoxy should not be a problem; however, if one takes into consideration that the temperature sensor embedded in the aluminum cage weighs 175 times more than a SAW strain gage, it is tempting to conclude that the behavior of the deviation at this temperature reflects the loss of bonding capability of the epoxy. As a general conclusion, it can be said that the error of a single temperature reading in rotation can be as high as 50, 75 or 83% at 25, 42 and 50 °C, respectively. On the other hand, Figure 11b shows the average temperature value calculated from the average frequency value of the array of readings (red data points). The values are much more stable. Readings at 25 and 42 °C show the same tendency and maximum absolute/relative errors of 5.2/20 and 4.8 °C/11%, respectively; the “deviation” from the trend line at ≈1800 rpm reinforces the idea of a mechanical vibration. Again, the average values taken at 50 °C show a more erratic behavior, even though the maximum absolute error is only 3.57 °C, with a corresponding relative error of 7%. In an attempt to decrease the influence of the large scattering of the measurements, the mean temperature was calculated again using only frequency readings that fall within the standard deviation—Figure 11b, red data points. The difference is more significant at 50 °C. This, together with the fact that the average temperature measured by the SAW sensor at 25 and 42 °C shows an apparent tendency to increase as rotation velocity decreases, suggests that the cell temperature might not have reached its final value when the measurements began.

These results fall far from the sub-Kelvin resolution obtained by Chrétien and his co-workers [23], who used a monopole antenna and not a capacitive coupler. In the current case, the lack of resolution is attributed to the instability of the link budget caused by the excessive vibration of the couplers, which, in turn, produces a large parasitic frequency shift with a negative impact in the accuracy of the measurements.

#### 3.4.2. Influence of the Rotation in the Torque Measurements

**Figure 12:**
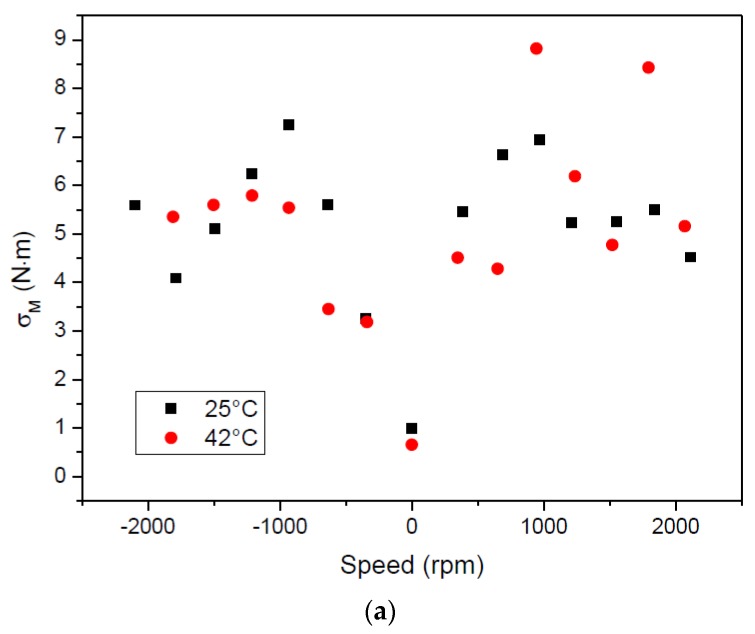

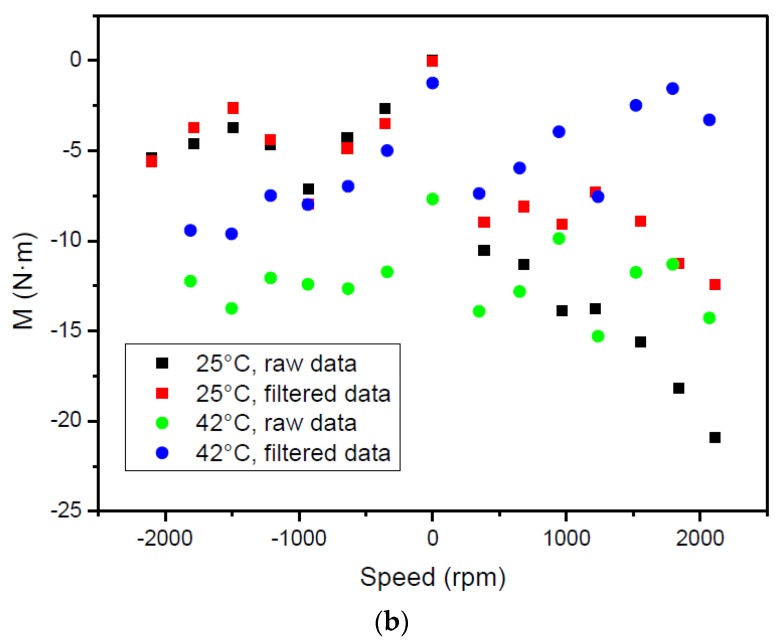
(**a**) Standard deviation and (**b**) torque measured at different rotation speeds. Black and green data points: values calculated with all frequency readings. Red and blue data points: values calculated with frequency readings that fall within the standard deviation.

Similarly to what was performed with the temperature sensor, 1000 frequency readings of both strain sensors were recorded and the measured torque was determined using Equation (10). The standard deviation of the array of torque readings is shown in Figure 12a. As the load cell begins to rotate, the standard deviation increases, but the effect of rotation speed is not quite clear since the values are scattered. The maximum values recorded were ≈7.2 and 8.4 N·m at 25 and 42 °C, respectively. The effect of rotation in the stability of the electromagnetic coupler is similar to the effects in measuring the temperature described in the previous section. However, there is an additional source of error related with the configuration of the SAW strain sensors. The W.FL connector is located at the extremity of the strain sensor; as a consequence, the centrifugal force felt by the cable “pulls” the sensor away from the load cell surface, making it behave like a cantilever and inducing the appearance of a measurable strain. This is more evident in Figure 12b (black and green data points) that shows the average value of the torque calculated from the average frequency readings. At 25 °C, the torque increases with rotation speed in a clockwise direction, reaching a maximum value of ≈−20.9 N·m. In the counterclockwise direction, the torque is more or less stable around −5 N·m. At 42 °C, the average value again stabilizes around −12.5 N·m. Measurements obtained at 65 °C are not shown since the W.FL cables became very soft and the electrical connection was too loose to give reliable signals. Similarly to what was done for the temperature, the average torque was calculated again using only the frequency readings that fall within the standard deviation—Figure 12b (red and blue data points). As a result, the absolute value of the calculated torque generically decreases; the maximum absolute values at 25 and 42 °C decrease from 20.9 (at 2100 rpm) and 15.3 N·m (at 1200 rpm) to 12.4 and 7.5 N·m, respectively. This reinforces the fact that the mechanical instability of the link budget, together with the cantilever effect introduced by the wires (which also vibrate during the rotation), are responsible for the low resolution of the measurements.

#### 3.4.3. Measurement of Torque in Rotation

**Figure 13:**
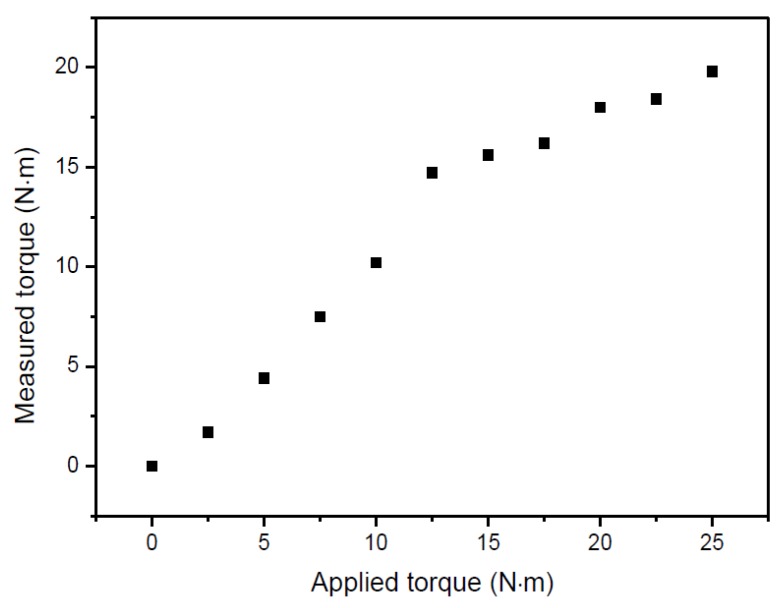
Torque measured by the strain sensors as a function of the torque applied by the braking system.

Given the problems that the strain sensors have at high rotation speeds (on one side the instability of the electromagnetic coupling, on the other side the cantilever effect induced by the W.FL cables, aggravated at high temperatures), the torque measurements under rotation were only performed at ambient temperature and at a low rotation speed. Figure 13 shows the torque measured by the strain sensors as a function of the torque applied by the braking system. For values of applied torque larger than 15 N·m, the deviation between the measured and applied torque value increases; this effect may be due to the heating of the braking pad and corresponding change in the friction coefficient, which deviates from the applied torque from the theoretical value. In addition, when the braking system is activated, a compressive force is applied to the shaft, which may reflect in the actual torque that is applied to the shaft and measured by the gages.

## 4. Discussion

The possibility of using SAW sensors to measure temperature and torque in the shaft of a power gearbox was evaluated with a COTS solution fabricated by SENSeOR. This system is composed of the interrogation unit WR D005, the temperature sensor TSE F162 and strain gages SSE E015 and SSE E017 mounted in half bridge configuration. The communication between the interrogation unit and the sensors can be made with coaxial cables or with two omnidirectional antennas. The temperature sensor was calibrated in a thermal chamber, while strain gages were calibrated using a custom-designed and fabricated mechanical setup. A second mechanical setup that allowed the application of controlled torque while the load cell was rotating was also fabricated in order to emulate a simplified gear box shaft.

The COTS solution (interrogation unit + sensors + antenna) can in principle be used without any need of adaptation to remotely measure temperature and strain/torque in any flat surface. The temperature sensor can be glued or fixed with screws; fixation by magnets in the metallic basis is also possible (http://www.senseor.com/senseor-monitoring-solutions/wireless-temperature-monitoring/tsa-h161-v). The accuracy of the temperature sensor (after proper calibration) is +/−1 °C. The strain sensors need to be glued to the surface of the part to be monitored with proper adhesive. The typical strain sensitivity at room temperature is 0.94 and −0.08 ppm/(µm/m) in the *x*- and *y*-direction, respectively), which leads to a gage factor higher than 3. Since the gage factor of a metallic extensometer is typically 2, SAW strain sensors have potentially higher precision and accuracy. If, however, the surface to be monitored is not flat (like for instance the surface of a shaft) a flat area needs to be machined before gluing the sensors; contrary to a flexible extensometer, for instance, SAW sensors are fabricated on a rigid piezoelectric material and, as such, do not bend. In [12], for instance, the surface of the shaft was processed before assembling the SAW sensors. In some cases, however, this procedure may make the shaft itself more fragile and would need to be avoided. One way to overcome this issue would be to clamp the temperature sensor to the shaft, like in [20]. In this case, the sensor would not be measuring the temperature of the shaft directly and the pre-calibration of the set sensor + clamping ring + shaft would be required. Following a similar approach, the SAW strain gages could also be glued on a plate transducer or on an outer ring previously designed to fit the curvature of the shaft. However, this would mean adding another interface so the transfer of strain between the shaft and the plate or ring transducers should be previously determined. In addition to the issues related with the assemblage of the sensors, it should be also taken into consideration that the Young’s modulus of the piezoelectric material is much higher than the one of adhesives that typically are used. Any asymmetries in the process of gluing different gages will have an impact in the transfer of strain from the part to be monitored to the sensor, especially at higher temperatures when the viscoelastic properties of the adhesive begin to change. The assemblage of the temperature sensor requires a circular 35.5 mm-diameter flat base (if attached to the antenna radome) or, alternatively, a ~26 × 16 mm^2^ rectangular flat base (to accommodate the aluminum cage). Since individual strain sensors are 9.0 × 5.4 mm^2^, half-bridge configuration requires at least a ~15 × 15 mm^2^ flat area. If both strain and temperature sensors are to be assembled, the required flat area is ~50 × 15 mm^2^ (temperature sensor attached to radome antenna) or ~26 × 32 mm^2^ (temperature sensor without antenna). After the sensors have been fixed on the surface to be monitored, the temperature sensor can be connected to the antenna by means of a 27 cm-long coaxial cable, while strain sensors can be connected with a 22 cm-long W.FL coaxial cable; this allows the antennas to be placed away from the sensors at a more convenient location, if necessary.

However, if the shaft is rotating, the COTS system is not adequate for immediate use and a series of constraints were identified:

1. Due to the large dimensions of the sensor antennas and to the difficulty to guarantee that sensors are in continuous line of sight with the interrogation unit, the antennas have to be replaced by a RF coupler. The mechanical stability of the RF coupler plays a crucial role in the stability and accuracy of the measurements with rotation speed. If, however, the variable changes slowly (like in the case of temperature or steady-state strain measurements), an array of several frequencies can be obtained; in this case, the value of the variable is calculated from the average frequency value and the accuracy of the readings improves considerably.
2. A second problem that arises is related with the sensors themselves. The temperature sensor comes embedded in an aluminum cage that cannot be removed and its weight (7 g) may unbalance the shaft at high rotation speeds. Both temperature and strain sensors have to be connected to the antenna by means of coaxial or W.FL coaxial cables. The mass of the cables cannot be neglected, nor can the centrifugal forces that appear when the shaft is rotating, which distort and pull the cables away from the sensors; this induces a strain on the piezoelectric material that compromises the accuracy of the readings. This effect is worse in the case of the strain sensors: since the W.FL connector is located in one of the extremities of the piezoelectric slab, the centrifugal force applied on the cables induces a cantilever effect that is an additional source of error.
3. The third problem is related with the acquisition rate: the user may define a confidence interval for the readings or even force the interrogation unit to average the results obtained during several interrogation windows. This is not problematic in the case of temperature readings, since temperature is a slow-changing magnitude. Strain, however, is expected to change rapidly and the accurate monitoring requires a large interrogation rate (a few tenths of even hundreds of Hz). If the configuration is made based on the confidence interval or on the window averaging, the interrogation rate may be seriously compromised.
4. The user does not have access to the full frequency response of the sensors. Like what was said above, the accuracy of the resonance frequency reading may be improved by defining a confidence interval of by averaging multiple readings. However, this is a limited procedure, since it relies in defining the parameters of the interrogation unit. It would be more useful if the user could have access to the full range of measured frequencies at each interrogation cycle; this way, it would be possible for the user to use different interpolation algorithms in order to improve the accuracy of the resonant frequency.

## 5. Conclusions

As a general conclusion, it can be said that the COTS system fabricated by SENSeOR is adequate to measure temperature and strain/torque of a piece at rest—as long as there is flat area where the sensors can be glued on. If this is not the case, the surface of the shaft needs to be previously processed; alternatively, clamping the sensors or adding a ring or plate transducer may be considered. The development of a COTS solution that could be used to monitor a rotating piece would involve the following steps:

1. Optimize the interrogation procedure in order to support higher acquisition rates, which would lead to a higher detail on the dynamics of rotational systems.
2. Increase the accuracy of the resonance frequency reading by using better control algorithms and, consequently, better interrogation algorithms, able to track the variation of the sensors resonance frequencies.
3. Give the user freedom to record the full range of measured frequencies at each interrogation cycle; this would give the user the freedom to post-process the frequency response and extract the resonance frequency with the desired accuracy.
4. Fabricate the SAW gage and temperature sensor dies without a W.FL connector. In this case, a small PCB board with the required W.FL connectors could be glued close to the sensors directly on the surface under probing. W.FL coaxial cables could be used to connect the coupler to the PCB. On the other side, the sensors could be electrically connected to the PCB by means of a short bond wire (for instance with a diameter of 25 µm). This would minimize the centrifugal force and the influence of the rotation on the measurements.
5. Design and implement a mechanically stable RF coupler.

## Figures and Tables

**Table 1:** Resonant frequencies at 25 °C, first and second order temperature coefficients of each resonator of TSE F162 temperature sensor and of SSE E015 and SSE E017 strain gages. Values taken from the sensors’ datasheets.

Sensor Reference	$f_{R_{0}}$ (MHz)	$C_{1}$ ($\times 10^{-6}$/°C)	$C_{2}$ ($\times 10^{-9}$/°C^2^)
TSE F162	435.90 ± 0.15	4.8	−24.6
	436.80 ± 0.15	−1.4	−33.1
SSE E015	433.97 ± 0.15	0.9	−24.8
SSE E017	433.32 ± 0.15	0.9	−24.8

**Table 2:** Length *L* and width *W* of the micro strip transmission lines calculated as a function of the thickness *H* of the FR-4 substrate.

Parameter	Rotor (λ4)			Stator (λ4)		
H (mm)	0.80	1.00	1.60	0.80	1.00	1.60
L (mm)	93.86	93.86	93.85	375.44	375.46	375.42
W (mm)	1.50	1.88	3.01	1.50	1.88	3.01

**Table 3:** Coefficients $C_{1}$ and $C_{2}$ obtained with dipole antennas and RF couplers. The theoretical values, obtained by replacing the coefficients taken from the sensor datasheet in Equation (4), are included for comparison.

Coefficients	Theoretical Values	Dipole Antennas	RF Couplers
$C_{1}$ ($\times 10^{-6}$/°C)	6.2	5.06	5.47
$C_{2}$ ($\times 10^{-9}$/°C^2^)	8.5	18.9	12.9

**Table 4:** Values of gage factor of strain gages alone and differential output obtained with force applied in the downward and upward directions.

Direction of Applied Force	$S_{G}$		
	SSE E015	SSE E017	Differential Output
Downwards	3.13	3.48	3.30
Upwards	3.00	4.31	3.66
